# How to Determine Accurate Conformational Ensembles by Metadynamics Metainference: A Chignolin Study Case

**DOI:** 10.3389/fmolb.2021.694130

**Published:** 2021-05-26

**Authors:** Cristina Paissoni, Carlo Camilloni

**Affiliations:** Dipartimento di Bioscienze, Università degli Studi di Milano, Milan, Italy

**Keywords:** molecular dynamics, metadynamics, metainference, statistical error, conformational ensembles

## Abstract

The reliability and usefulness of molecular dynamics simulations of equilibrium processes rests on their statistical precision and their capability to generate conformational ensembles in agreement with available experimental knowledge. Metadynamics Metainference (M&M), coupling molecular dynamics with the enhanced sampling ability of Metadynamics and with the ability to integrate experimental information of Metainference, can in principle achieve both goals. Here we show that three different Metadynamics setups provide converged estimate of the populations of the three-states populated by a model peptide. Errors are estimated correctly by block averaging, but higher precision is obtained by performing independent replicates. One effect of Metadynamics is that of dramatically decreasing the number of effective frames resulting from the simulations and this is relevant for M&M where the number of replicas should be large enough to capture the conformational heterogeneity behind the experimental data. Our simulations allow also us to propose that monitoring the relative error associated with conformational averaging can help to determine the minimum number of replicas to be simulated in the context of M&M simulations. Altogether our data provides useful indication on how to generate sound conformational ensemble in agreement with experimental data.

## Introduction

Molecular dynamics simulations (MD) are a powerful tool to study at high resolution the dynamics of biomolecules in solution, yet they rely on the quality of the physical model used to describe molecules (i.e., the force field) as well as on the computing power needed to acquire longer and longer trajectories that is better and better statistics (Bottaro and Lindorff-Larsen, 2018; Grossfield et al., 2019). Force fields have been dramatically improving in the last years and computing power is always increasing allowing to study more and more complex systems (Best et al., 2014; Huang et al., 2017; Robustelli et al., 2018). To further improve the extent of the sampling and the accuracy of the physical model, enhanced sampling techniques (Sugita and Okamoto, 1999; Laio and Parrinello, 2002) as well as techniques to integrate experimental data in MD have been developed (Fennen et al., 1995; Bonomi et al., 2016; Köfinger et al., 2019). Reviewing the vast literature on both topics is outside the scope and space of the present work and excellent reviews are available (Spiwok et al., 2015; Allison, 2017; Bonomi et al., 2017; Bottaro and Lindorff-Larsen, 2018; Camilloni and Pietrucci, 2018; Bernetti et al., 2020). Among these methods we have contributed to develop Metadynamics Metainference (M&M) (Bonomi et al., 2016a) that is a combination of Metadynamics (Laio and Parrinello, 2002), a popular enhanced sampling technique, and Metainference (Bonomi et al., 2016), a Bayesian scheme that allows for the integration of equilibrium experimental observables as restraints over multiple replicas of a simulation. M&M has been applied to combine different experimental observables and to work on a large variety of systems (Löhr et al., 2017; Eshun-Wilson et al., 2019; Heller et al., 2020; Jussupow et al., 2020).

**FIGURE 1:**
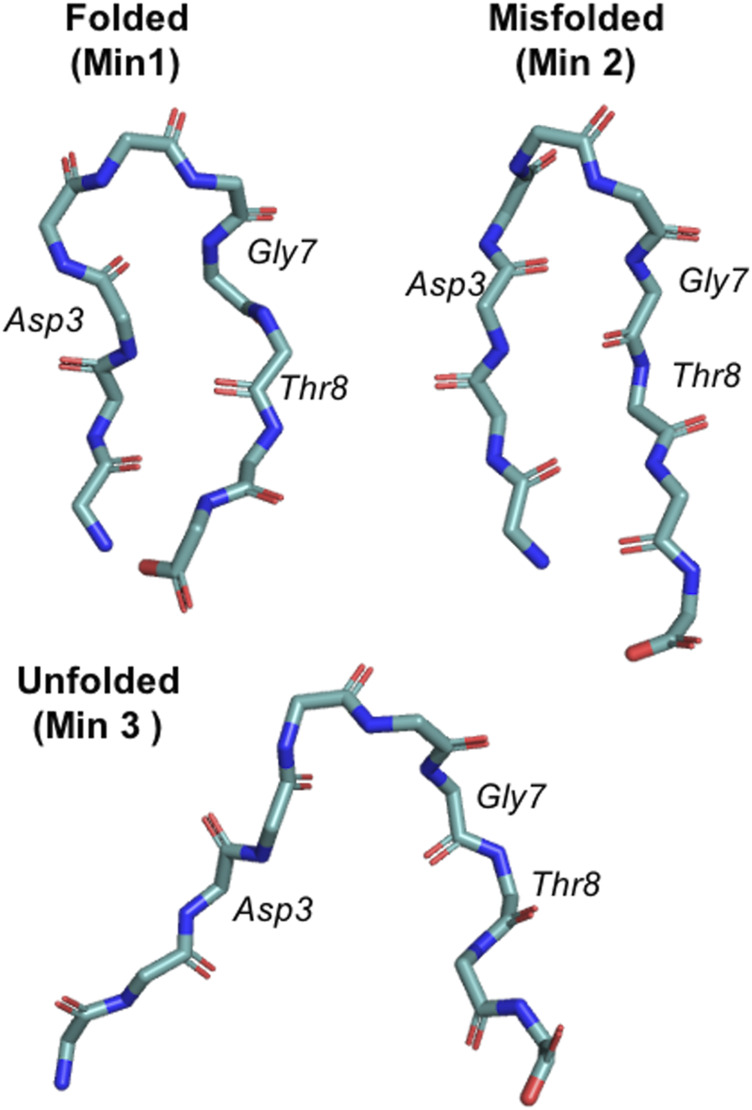
Representation of the three main chignolin minima corresponding to the folded (Min 1), misfolded (Min 2) and unfolded (Min 3) states.

In this work we aim to understand how Metadynamics should be ideally coupled to Metainference in order to guarantee optimal statistical precision and experimental accuracy. Multiple MetaD variants are available and M&M has always been coupled with Parallel Bias Metadynamics (PBMetaD), a variant specifically designed to enhance the sampling along many one-dimensional collective variables (CVs) (Pfaendtner and Bonomi, 2015). In particular we identified three key questions: 1) how reliable are the error estimates resulting from Metadynamics simulations when using a standard technique as block averaging (Flyvbjerg and Petersen, 1989); 2) how does multiple-walkers PBMetaD compare to conventional multiple-walkers MetaD and what are their pros-and-cons; 3) how do the two approaches combine with Metainference to achieve at the same time an optimal sampling and an optimal integration of experimental data? Of note, the first two questions apply not only to M&M but to the sound application of enhanced sampling techniques. To answer these questions, we investigated thoroughly the conformational space of chignolin (Figure 1), a 10 residues peptide that can populate three states and whose complexity, while not comparable to that of full-length proteins, is definitely greater than the widely used alanine dipeptide in vacuum (Kührová et al., 2012). In doing so we introduced a scheme to combine simple CVs into more complex ones with the aim of discriminating some identified reference states. By performing PBMetaD simulations with many simple CVs (PB20), PBMetaD simulations with less, optimally combined, CVs (PB4); as well as MetaD simulations with the same optimally combined CVs (ME2), all in triplicate (Table 1), we show that, 1) block-averaging provides a robust estimate of statistical errors; 2) PBMetaD and MetaD dramatically decrease the effective number of frames collected by MD and this effect is worse in MetaD. This second effect is very relevant in combining Metadynamics with Metainference because it decreases the number of effective replicas that can actually contribute to the estimation of the conformational heterogeneity associated with experimental observables. To test this effect, we then performed (Table 1) M&M simulations using either PBMetaD or MetaD and 10 or 100 replicas. To avoid effects related to the quality of the experimental data and the forward model, synthetic SAXS data have been obtained using as a reference a 40 μs long simulated tempering simulation of chignolin by Piana et al., 2020. Our results indicate that the minimum number of replicas in M&M simulations can be set by monitoring the relative error associated with the averaging of back calculated observables, and that this number is affected not only by the system and the calculated observable but also by the details of the Metadynamics setup.

**TABLE 1:** Summary of the simulations performed or analyzed in this work.

Name	Replicates	#Replicas	Enhanced sampling technique	#CV	Force field	Replica length (total) μs	Color code
Reference^a^	1	1	Simulated tempering	NA	DES-amber	40	Dark grey
PB20	3	10	PBMetaD	20	DES-amber	1 (30)	Blues
PB4	3	10	PBMetaD	4	DES-amber	1 (30)	Greens
ME2	3	10	MetaD	2	DES-amber	1 (30)	Violets
Prior	1	10	PBMetaD	4	99sb-ildn	1 (10)	Light grey
PB4(10r)	1	10	PBMetaD	4	99sb-ildn + M&M	0.5 (5)	Cyan
ME2 (10r)	1	10	MetaD	2	99sb-ildn + M&M	0.5 (5)	Yellow
PB4(100r)	1	100	PBMetaD	4	99sb-ildn + M&M	0.5 (50)	Blue
ME2 (100r)	1	100	MetaD	2	99sb-ildn + M&M	0.5 (50)	Orange

For each simulation are reported: the number of replicates, the replicas (or walkers), the enhanced sampling technique employed, the number of CVs, the force field, the length of each replica (and the total simulation time) and the color code associated to the simulation in the figures.

a This simulation was performed by Piana et al. (2020).

## Materials and Methods

### Molecular Dynamics Simulations of Chignolin

Simulations of chignolin were performed using GROMACS 2019 (Abraham et al., 2015) and PLUMED 2 (Tribello et al., 2014). In the first round of simulations the DES-amber force field (Piana et al., 2020) was used in combination with the tip4p water model with increased dispersion (Piana et al., 2015). A starting model of CLN025 chignolin was taken from PDB 5AWL (Honda et al., 2008) and solvated with 2,553 water molecules in a dodecahedron box initially 1.4 nm larger than the protein in each direction. The system was neutralized with a salt concentration of 100 mM NaCl. After an initial energy minimization to a maximum force of 100 kJ/mol/nm, the solute was equilibrated under NVT condition at the temperature of 340 K for 50 ps using the Berendsen thermostat (Berendsen et al., 1984); then Berendsen barostat was used to equilibrate the system in the NPT ensemble to the target pressure of 1 atm for 200 ps, maintaining the temperature at 340 K with the Bussi thermostat (Bussi et al., 2007). The equilibration phase was followed by an initial MD simulation of 250 ns, from which a pool of conformations was extracted to be used as starting models for the subsequent runs (run 1). Starting points for replicates run 2 and run 3, where instead extracted from run 1 thus resulting in very different initial conditions. The production runs were all performed in the NPT ensemble, maintaining temperature and pressure at the values of 340 K and 1 atm respectively, using the Bussi thermostat (Bussi et al., 2007) and the Parrinello-Rahman barostat (Parrinello and Rahman, 1981). Electrostatic was treated by using the particle mesh Ewald scheme (Essmann et al., 1995) with a short-range cutoff of 0.9 nm and a Fourier grid spacing of 0.12 nm; van der Waals interaction cutoff was set to 0.9 nm. For these simulations the hydrogen mass repartitioning scheme (Hopkins et al., 2015) was used to reduce the computational cost: the mass of heavy atoms was repartitioned into the bonded hydrogen atoms using the *heavyh* flag in the pdb2gmx tool; the LINCS algorithm (Hess et al., 1997) was used to constraint all bonds, eventually allowing to use a time step of 5 fs.

Using this set-up, we ran three different Metadynamics simulations, each performed in triplicates (named run 1, run 2, run 3, starting from different set of conformations). These are:

1. PB20: in which PBMetaD was employed and 20 CVs were biased. These include the phi/psi dihedral angles of the 10 amino acids composing chignolin (18 CVs), the gyration radius and the antiparallel beta sheet-content.
2. PB4: in which PBMetaD was employed biasing 4 CVs, comprising the gyration radius, the antiparallel beta sheet-content and 2 CVs optimized based on the knowledge of the folded, misfolded and unfolded chignolin conformations (named *back* and *cmap*, and based on a combination of backbone dihedral angle and of contacts between groups of atoms, see next section).
3. ME2: in which MetaD was employed using 2 CVs, the gyration radius and the optimized *cmap* collective variable.

All the simulations were performed adopting the multiple-walker scheme (Raiteri et al., 2006), simulating 10 replicas (or walkers): each replica was evolved for 1 µs, resulting in a 10 μs sampling per each simulation. Metadynamics was used in its well-tempered version (Barducci et al., 2008), where Gaussians with an initial height of 0.5 kJ/mol were deposited every 1 ps using a bias factor of 10. For all the CVs, the width of the Gaussians was determined with the dynamically adapted geometry-based Gaussian approach (Branduardi et al., 2012), using 0.015 nm as the extent of Cartesian space covered by a variable to estimate CVs fluctuations, and setting a minimum value for the width specific for each CV (0.03 rad for the dihedral angles, 0.004 nm for the gyration radius, 0.02 for the antiparallel beta sheet-content, 0.01 and 0.001 for the *back* and *cmap* optimized CVs).

Each simulation was analyzed by creating a concatenated trajectory and reweighting each frame by using the final Metadynamics bias potential, assuming a constant bias during the entire course of the simulation (Branduardi et al., 2012). To assess the convergence of the simulations and the associated statistical errors we used block-average analysis (Flyvbjerg and Petersen, 1989; Bussi and Tribello, 2019). According to this technique, the trajectory is split into a set of *NB* blocks of equal length. By comparing the averages of a given quantity from each block we can calculate the error bar on our estimate of that quantity: for large enough blocks the averages should not be time correlated so that the estimate of the error converges. As our blocks could be characterized by different weights, this must be taken into account in the estimation of the error as described in (Invernizzi et al., 2020). Given *W*_*b*_ the weight of the block *b*, obtained as the sum of the weights of the frames composing the block, the statistical error on the observable *O* is: $err_{O}=\sqrt{\frac{1}{\left(NB_{eff}-1\right)}\frac{\sum_{b=1}^{NB}W_{b}\;{\left[{\hat{O}}_{b}-\hat{O}\right]}^{2}}{\sum_{b=1}^{NB}W_{b}}}$, where $NB_{eff}={\left(\sum_{b=1}^{NB}W_{b}\right)}^{2}/\sum_{b=1}^{NB}W_{b}^{2}$ is the effective block size, the sums run on the number of blocks NB, ${\hat{O}}_{b}$ is the average computed over the frames of block b and $\hat{O}$ is the average computed over all the frames, which corresponds to the average computed over the block averages, i.e. $\hat{O}=\sum_{b=1}^{NB}W_{b}\;{\hat{O}}_{b}/\sum_{b=1}^{NB}W_{b}$. As pointed out in (Invernizzi et al., 2020) when the weights of the blocks are unbalanced, using NB instead of $NB_{eff}$ can significantly underestimate the uncertainty.

### Optimized Collective Variables

PBMetaD can in principle bias many CVs using one-dimensional Gaussians (Pfaendtner and Bonomi, 2015), but often these CVs are simple in nature (like dihedrals or distances) thus losing the complex correlations that may be at play in slow reaction coordinates. Finding optimal CVs is a complex problem that requires the previous knowledge not only of the different states but also of the pathways connecting them. Example of methods using reactive pathways to estimate optimal CVs include TICA, SGOOP and machine learning approaches (Tiwary and Berne, 2016; McCarty and Parrinello, 2017; Sultan and Pande, 2017, 2018; Wang and Tiwary, 2020). Instead of learning from reactive pathways one can instead try to only maximize the discrimination of the different states as implemented in HLDA (Mendels et al., 2018). One possible limitation of this latter approach, which has the clear advantage of being more affordable for large and complex systems, is that a CV that optimally discriminate states may not correspond to an efficient reaction coordinate. Here we propose a simple method to generate a novel $CV\left(a,\varphi \right)=\sum_{i=1}^{N}a_{i}\varphi_{i}$, where ***a*** is a normalized vector of size *N*, starting from *N* input simple collective variables ***φ*** (e.g., these could be the backbone dihedral angles, or the C⍺-C⍺ contacts). CV(a,φ) while trying to discriminate two or more states, tries also to 1) discard as few of the input CVs ***φ*** as possible by keeping the weights ***a*** of the combined CVs as uniform as possible; and 2) keep the width of the minima comparable. This latter property is relevant for methods like Metadynamics that uses Gaussians. To achieve these properties the optimal value a is obtained by minimizing the following scoring function (here given for two states indicated as 1 and 2):

$$\psi \left(a\right)=-\frac{<CV_{1}>-<CV_{2}>}{2\left(\sigma_{CV_{1}}^{2}+\sigma_{CV_{2}}^{2}\right)}+\frac{\max\left(\sigma_{CV_{1}},\sigma_{CV_{2}}\right)}{\min\left(\sigma_{CV_{1}},\sigma_{CV_{2}}\right)}+\sum_{i=1}^{N}a_{i}^{2}\ln\frac{a_{i}^{2}}{1/N}$$

where the first term maximizes the discrimination among states, the second keeps the width of the minima comparable, the last keeps the parameters as uniform as possible.

This approach is then applied to optimize two CVs, *back* and *cmap*, as the combination of chignolin backbone dihedral angles and the contacts among the center of the backbone of i − i + 3 aminoacids, respectively. The CVs are first calculated for the three states as observed in the preliminary 250 ns long simulation (Supplementary Figure S1) and then their combination is obtained as described above. The distribution of the values for the *cmap* CV before and after optimization is reported in Supplementary Figure S1.

### Metainference

Metainference is a technique based on Bayesian inference and replica-averaging modeling (Rieping, 2005; Cavalli et al., 2013; Bonomi et al., 2016). Following the replica-averaging modeling strategies, multiple replicas of the system are simulated in parallel and the quantities to be restrained against experimental data are back-calculated as averages over the replicas, thus taking into account the effects of conformational averaging. Bayesian inference allows to modulate the strength of the restraints estimating, along with the model, statistical errors, which include random and systematic errors as well as inaccuracies of the forward model.

In the case of Gaussian noise, the Metainference energy is described by (Löhr et al., 2017): $E_{MI}=E_{FF}+\frac{k_{B}T}{2}\sum_{i=1}^{N_{d}}\sum_{r=1}^{NR}{\left[d_{i}-\lambda \langle f_{i}\left(X\right)\rangle \right]}^{2}/{\left(\sigma_{r,i}^{B}\right)}^{2}+{\left(\sigma_{i}^{SEM}\right)}^{2}+E_{\sigma }$, where $E_{FF}$ is the force field energy, $k_{B}$ is the Boltzmann constant, *T* the temperature, *d* the set of $N_{d}$ experimental data, *f*(***X***) is the forward model used to back-calculate the observable from conformation ***X***, $f_{i}\left(X\right)$ indicates the average over the NR replicas for observable *i*, $\sigma_{r,i}^{B}$ is an uncertainty parameter that describes random and systematic errors, $\sigma_{i}^{SEM}$ is the standard error of the mean related to conformational averaging,  λ is an optional scaling parameter and $E_{\sigma }$ is an energy term that accounts for normalization of the data likelihood and error priors. In Metainference Monte Carlo sampling is used to sample both the uncertainty $\sigma_{r,i}^{B}$ (which depends on both the replica and the observable) and optionally the scaling parameter *λ*.

Metainference can be combined with Metadynamics (M&M) to accelerate the exploration of the conformational space (Bonomi et al., 2016; Löhr et al., 2017). In M&M the replicas share the Metadynamics bias potential as in the case of multiple-walkers method (Raiteri et al., 2006). Depending on the bias potential *V*_*G*_ each replica *r* has a different weight that can be approximated on the fly as $w_{r}∼e^{V_{G}\left(CV\left(X_{r}\right)\right)/k_{B}T}$, with *CV*(***X***_*r*_) representing the set of selected CVs, functions of the microscopic coordinates ***X***. Therefore, these weights must be taken into account when calculating the experimental averages and the standard error of the mean $\sigma_{i}^{SEM}$, that are computed as: $f_{i}\left(X\right)=\sum_{r=1}^{NR}w_{r}f_{i}\left(X_{r}\right)/\sum_{r=1}^{NR}w_{r}$ and $\sigma_{i}^{SEM}=\sqrt{\frac{1}{\left(NR_{eff}-1\right)}\;\frac{\sum_{r=1}^{NR}w_{r}\;{\left[f_{i}\left(X_{r}\right)-\langle f_{i}\left(X\right)\rangle \right]}^{2}}{\sum_{r=1}^{NR}w_{r}}}$, with $NR_{eff}={\left(\sum_{r=1}^{NR}w_{r}\right)}^{2}/\sum_{r=1}^{NR}w_{r}^{2}$ representing the number of effective replicas. In order to reduce the noise resulting from the instantaneous fluctuations of the bias, the weight of each replica is calculated *via* a moving average of the bias over a given number of MD steps (set by the keyword AVERAGING). Also, to reduce the oscillations of $\sigma_{i}^{SEM}$ we used the maximum value of $\sigma_{i}^{SEM}$ over the same time window defined by AVERAGING keyword. Finally, we automatically determined the maximum values that can be sampled for $\sigma_{r,i}^{B}$ as $\max\left(\sigma_{r,i}^{B}\right)=\sigma_{i}^{SEM}\sqrt{NR}$, with NR being the number of replicas (this option can be set in plumed using the keyword OPTSIGMAMEAN = SEM_MAX).

### Small-Angle X-Ray Scattering (SAXS)-Driven Molecular Dynamics Simulations

Synthetic SAXS intensities, to be used as target for the restraints in our simulations, were calculated from a reference 40 μs long MD trajectory, performed with the DES-amber forcefield and provided by Piana et al. 2020. From this simulation a set of 24 representative SAXS intensities at different scattering angles, ranging between 0.01 and 1.39 Å^−1^ and equally spaced, were calculated with PLUMED using atomistic structure factors and considering only the trajectory frames with temperature close to 340 K (Paissoni et al., 2019; Paissoni et al., 2020). While we know that this range is not representative of a realistic SAXS experiment, considering the small dimension of the protein we decided to use such a large range to include higher resolution details. SAXS restraints were applied every 2 MD steps and atomic scattering factors were used to back-calculate the 24 SAXS intensities. The SEM_MAX option was used to automatically estimate both the $\sigma_{i}^{SEM}$ as well as the maximum value of $\sigma_{r,i}^{B}$ for the M&M simulations; the window averaging for the estimation of the weights was performed on a time window of 1 ps to match the frequency of deposition of Metadynamics hills.

For the set of SAXS-driven simulations we used as prior the amber99sb-ildn (Lindorff-Larsen et al., 2010) force field with the tip3p water model (Mark and Nilsson, 2001). The system was prepared and equilibrated as described above and a set of starting conformations was generated from a 1 μs long plain MD simulation. We performed five Metadynamics simulations (Table 1): one unrestrained, prior, amber99sb-ildn simulation, using the PB4 setup with 10 replicas; two simulations, PB4 (10r) and PB4 (100r), with the PB4 setup plus SAXS restraints using either 10 or 100 replicas; two simulations, ME2 (10r) and ME2 (100r), with the ME2 setup plus SAXS restraints, using either 10 or 100 replicas. The unrestrained prior simulation evolved for 1 μs per replica, while the SAXS driven simulations evolved for 500 ns per replica.

The input files for all the simulations of this work are deposited in PLUMED-NEST (The PLUMED Consortium, 2019) as plumID:21.014.

## Results

Metadynamics and M&M simulations, using either PBMetaD or conventional MetaD, were performed to understand: 1) the statistical precision achievable by different Metadynamics setups; 2) the role played by enhanced sampling in the integration of experimental information in MD simulation by Metainference.

### Assessing the Statistical Precision of Metadynamics Simulations

**FIGURE 2:**
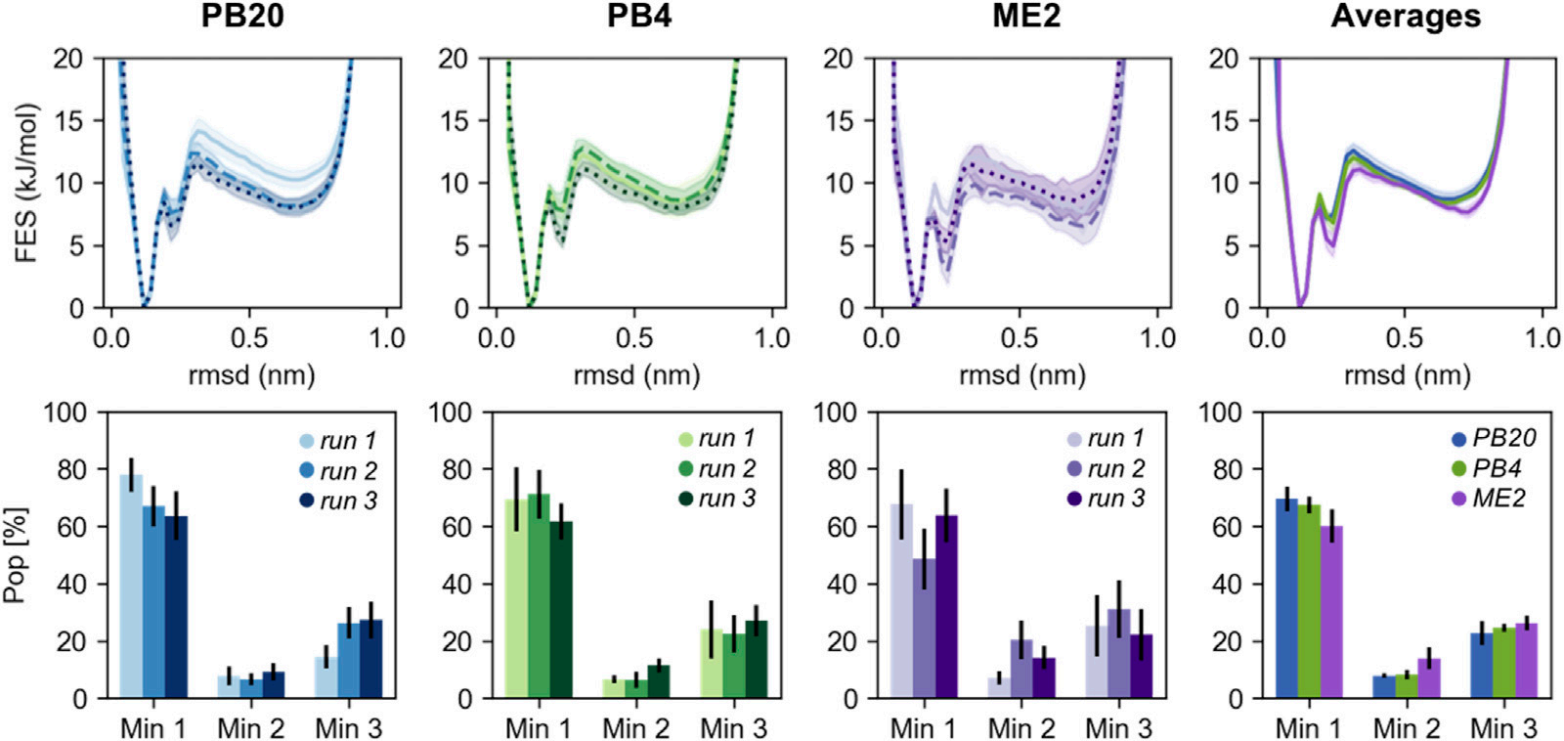
The RMSD free energies **(top panels)** and the population of the three main chignolin minima **(bottom panels)** are represented for different sets of simulations (PB20 in blues, PB4 in greens, ME2 in violets shades). The different shades indicate each of the runs of the triplicates and the errors are estimated *via* block-average analysis. In the rightest panels are reported the averages computed over the triplicates for each set of simulations; here the errors are determined as standard errors over the triplicates as described in the main text. In all the pictures the free energies are shifted to set their minimum to 0.

PBMetaD or conventional MetaD, was used to simulate the folding and unfolding of chignolin close to the transition temperature and to compute the free energy and the equilibrium population related to its three main conformational states (Figure 1). In particular we focused our attention on the ability to correctly estimate the errors associated to these calculations. Estimating statistical errors in enhanced sampling MD of large systems is a relevant problem because of their high computational cost. Previous works have already noted the importance of running multiple replicates, alternatively block-averaging can be used to estimate errors taking into accounts the time-correlated nature of MD. Here we compare statistical errors estimated from replicates with those resulting from block-averaging. In Figure 2 we rebuilt a free-energy profile as function of an unbiased collective variable, the RMSD (computed over the main chain plus the Cβ atoms) with respect to a reference folded state of chignolin, and we estimated the population of three minima: folded (Min 1, RMSD ≤1.9 Å), misfolded (Min 2, 1.9 Å ≤ RMSD ≤3.0 Å) and unfolded (Min 3, RMSD >3.0 Å, see Figure 1). The error of each simulation is estimated using block-averaging. Furthermore, averages and errors are obtained by the triplicates, where the average free energy of bin b is computed as $F_{b}=-k_{B}T\log\left(p_{b}\right)$ and the associated errors are estimated as $err_{F_{b}}=\frac{1}{\sqrt{3}}k_{B}T\frac{\sigma_{p_{b}}}{p_{b}}$, with $p_{b}$ being the average probability of the bin computed over the triplicates and $\sigma_{p_{b}}$ its standard deviation.

Qualitatively, the resulting free energies display a good overlap both within the triplicates and when comparing the three simulation setups (Figure 2). Major deviations are mainly located in the high energy regions (>*2k*_*B*_ *T*). Nevertheless, we note that the variability among simulations strongly affect the population of the three minima, leading to differences for the folded minimum from less than 10% for PB4 simulations to ∼20% for ME2 simulations. The populations estimated by averaging over the replicates are more precise and in quantitative agreement among the three simulation setups stressing once again the importance of running independent simulations. Reassuringly, errors calculated by block-averages (comparing the free-energy obtained from blocks of lengths in the range 30 ns–1 µs), correctly estimate the variability observed within the triplicates (Figure 2), with ME2 simulations showing the largest error in the set. Free-energies and errors estimated as a function of the other biased and unbiased CVs (Supplementary Figures S2, S3) display a consistent behavior. Furthermore, we compared our results with a reference 40 μs long simulated tempering simulation published in Piana et al. (2020), showing that the populations of the minima are quantitatively in agreement with those obtained averaging over our replicates (Supplementary Figure S4).

To rationalize the higher variability observed in ME2 simulations with respect to the PB20 and PB4 simulations, we calculated the number of transitions between the folded and unfolded state as well as the effective statistics, i.e., the fraction of frames actually contributing to our statistical observations (cf. Table 2). While the number of transitions per microsecond is slightly lower in ME2 with respect to PB20 and PB4, the effective number of frames is surprisingly low for all simulations and dramatically so for ME2 (Table 2). This is likely due to the wider exploration of the conformational space by MetaD, that spends more time in high free-energy regions, thus reducing the fraction of frames that actually populate the most relevant conformations (see also Supplementary Figure S5). This reminds us that enhanced sampling is not a free lunch: indeed, while favoring the exploration of a wider conformational space, it reduces the statistical precision of the low free-energy regions reconstruction. A similar observation can explain the difference in the effective frames observed between PB20 and PB4. To improve the statistics one possibility is to fine tune and decrease the bias factor employed for well-tempered Metadynamics (here it was 10 for all setups, a very common value for simulations of biological molecules) and thus focus the sampling only within regions of interest.

**TABLE 2:** For each replicate of the PB20, PB4, and ME2 simulations are reported: 1) the number of transitions per microsecond from the folded (F) to the unfolded (U) state and vice versa; 2) the percentage of the effective frames, *NF*_*eff*_, over the total number of frames (*NF*).

		Transition per μs		NF_eff_/NF (%)
		U -> F	F -> U	
PB20	Run 1	2.2 ± 0.4	2.8 ± 0.3	37%
	Run 2	2.2 ± 0.4	1.9 ± 0.3	39%
	Run 3	1.9 ± 0.3	1.8 ± 0.3	39%
	Average	2.1 ± 0.1	2.2 ± 0.3	38 ± 0 1%
PB4	Run 1	2.0 ± 0.4	2.8 ± 0.5	22%
	Run 2	2.1 ± 0.4	1.8 ± 0.4	20%
	Run 3	2.2 ± 0.4	2.5 ± 0.5	26%
	Average	2.1 ± 0.1	2.4 ± 0.3	23 ± 0 2%
ME2	Run 1	1.6 ± 0.6	2.4 ± 0.5	2.7
	Run 2	1.2 ± 0.4	1.3 ± 0.4	4.1
	Run 3	1.1 ± 0.3	1.4 ± 0.4	3.0
	Average	1.3 ± 0.2	1.7 ± 0.4	3.3 ± 0.4%

*NF*_*eff*_ is computed as: $NF_{eff}={\left(\sum_{i=1}^{NF}w_{i}\right)}^{2}/\sum_{i=1}^{NF}w_{i}^{2}$, where $w_{i}$ is the weight associated to each frame. The average and the standard error over the triplicates are also reported.

### Metadynamics Metainference: Enhanced Sampling and Conformational Averaging

The poor statistics characterizing our Metadynamics simulations, and ME2 in particular, raises issues about their combination with Metainference, in particular when the experimental data to be integrated represent averages over multiple conformational states. To test this effect, we performed 4 M&M simulations with the amber99sb-ildn force field, using as restraints synthetic SAXS data derived from reference 40 μs long DES-amber trajectory. The choice of SAXS is due to the ability of this technique to capture the overall size and shape of the molecules, thus being particularly sensitive to the equilibrium between the different conformational states (see Supplementary Figure S6); herein the use of synthetic data allows to avoid experimental and forward model errors and to focus on the effect of Metadynamics on the number of effective replicas.

We firstly performed a prior 10 replicas PB4 simulation, with the amber99sb-ildn force field, verifying that the resulting conformational ensemble and the back-calculated SAXS profiles are significantly far from the reference DES-amber simulation (Supplementary Figures S7, S8). Then we tested four different SAXS-restrained M&M setup, either using PB4 or ME2 with 10 or 100 replicas (Table 1). The inclusion of SAXS restraints improve, as expected, the agreement with the input scattering profile $I_{ref}$. We found that the relative error of the calculated SAXS intensity, defined as $R_{factor}=\left|\frac{I-I_{ref}}{I_{ref}}\right|\times 100$, is in the range 0.4–1.0% (Supplementary Figure S9), representing a significant improvement with respect to the prior amber99sb-ildn simulations (*R*_factor_ = 6.7%, Supplementary Figure S8). Also, we observe that in all the cases the input profile is well in agreement with the one back calculated from the simulations within the error estimated by Metainference (Supplementary Figure S9). Nevertheless, it is worth noting that the estimated errors differ in the four simulations as it will be discuss later, thus slightly impacting the extent of the agreement with the input data: i.e., larger errors result in slightly worse agreement as in ME2 (10r), while smaller errors lead to better agreement as in PB4 (100r).

**FIGURE 3:**
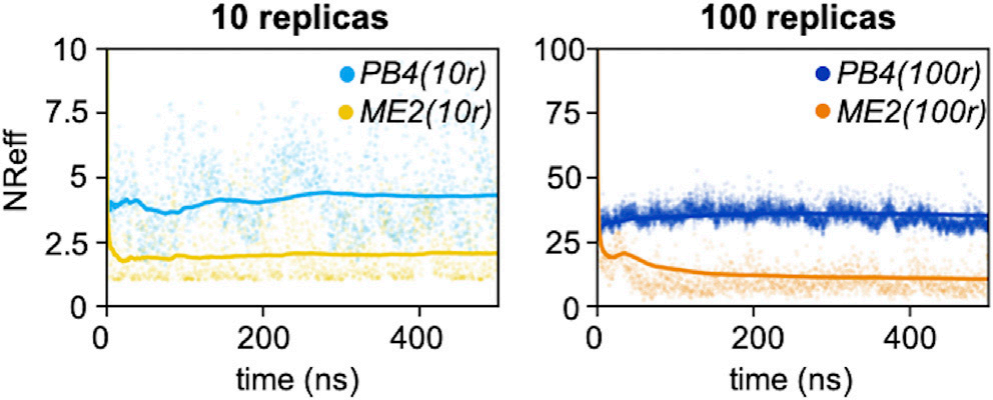
Number of effective replicas as a function of the simulation time for PB4 (10r), ME2 (10r), PB4 (100r) and ME2 (100r) simulations. The dots represent the value at the exact time, while the straight lines indicate the cumulative averages.

**FIGURE 4:**
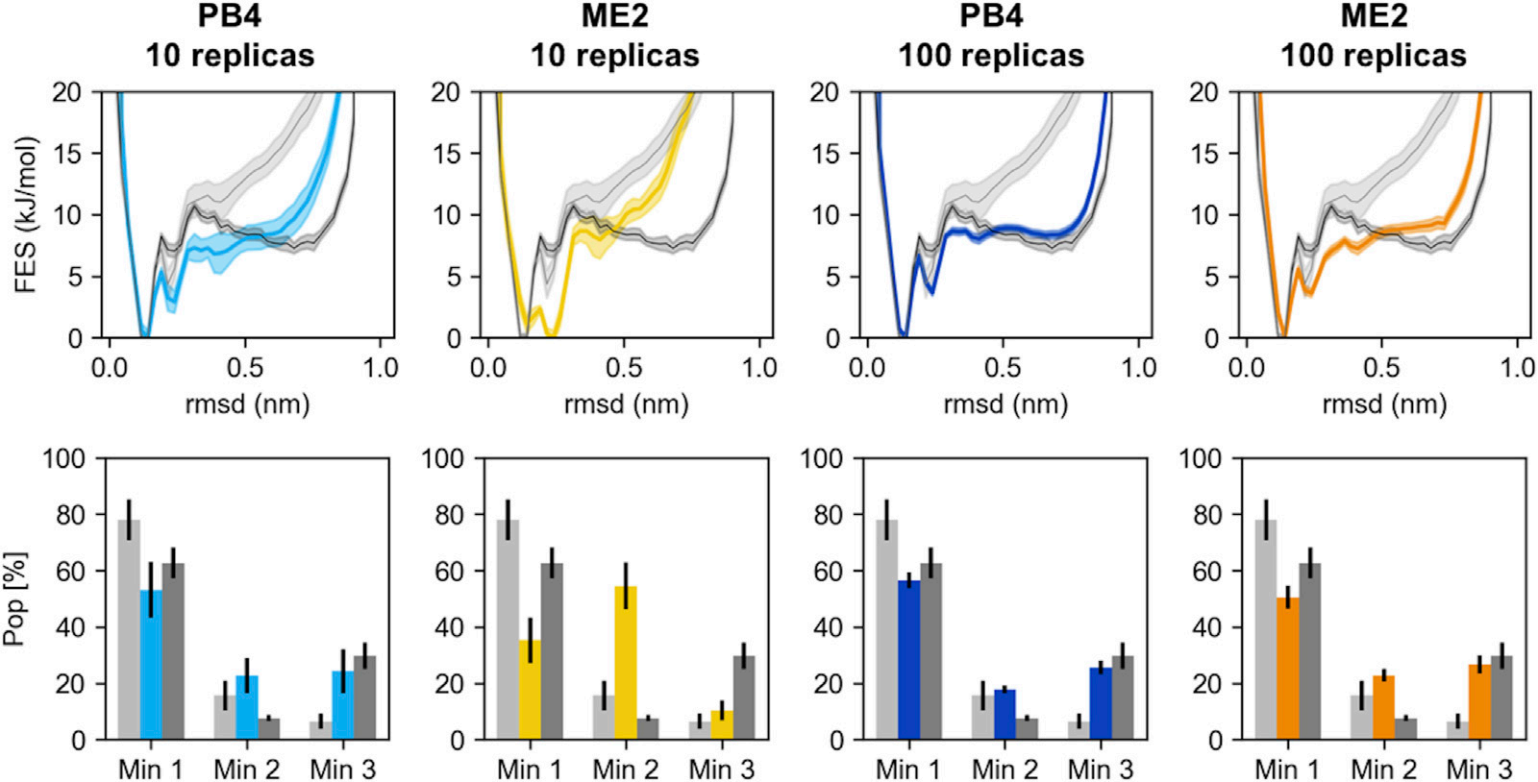
The RMSD free energies **(top panels)** and the population of the three main chignolin minima **(bottom panels)** are represented for different simulations (PB4 or ME2, with either 10 or 100 replicas). The results from the prior simulation, performed with the amber 99sb-ildn force field and no SAXS restraints, are represented in light grey. The results from the reference simulation, performed with the DES-amber force field and whose back-calculated SAXS intensities were used as target, are represented in dark grey. The errors are estimated via block-average analysis; the free energies are shifted to set their minimum to 0.

Next, for each of the four simulations we monitored the number of effective replicas as a function of the simulations time. With the same number of actual replicas, the PB4 setup displayed more effective replicas than ME2 (Figure 3): the average *NR*_*eff*_ in PB4 (10r) was two times larger, 4.3, than in ME2 (10r), 2.0, and it was more than three times larger in PB4 (100r) than in the ME2 (100r) setup (*NR*_*eff*_ of 35 vs. 10). This difference impacts on the resulting conformational ensemble (Figure 4; Supplementary Figure S10). A striking effect is seen for the ME2 (10r) simulation (*NR*_*eff*_ = 2.0), in which the inclusion of SAXS data caused a strong distortion of the original ensemble leading to the formation of a new main minimum and a clear deviation from the target also in the low free-energy regions. This is consequence of the fact that, in time, we are forcing approximately *NR*_*eff*_ = 2.0 conformations to fit SAXS data that can only be explained by larger conformational ensembles. Importantly, the reconstructed ensembles become increasingly close to the target for larger values of *NR*_*eff*_, with the best agreement obtained for PB4 (100r) (*NR*_*eff*_ = 35). We observe that this does not mean that PB4 allows a better agreement than ME2 in general, but it suggests that to obtain a comparable agreement more replicas are needed when using the ME2 setup.

The number of effective replicas also affect the model errors sampled by Metainference. As expected, we observed a direct effect on $\sigma_{i}^{SEM}$ (Supplementary Figure S11), where less effective replicas resulted in larger errors: indeed $\sigma_{i}^{SEM}$ represents the uncertainty related to conformational averaging, which is consequence of the fact that we are using a small number of conformations (*NR*_*eff*_) to back calculate experimental data, that are ideally obtained as averages over an infinite number of conformations. We also noted an indirect effect on $\sigma_{r,i}^{B}$, where again fewer effective replicas resulted in larger errors (Supplementary Figure S11). This is likely due to a better agreement with input data for larger *NR*_*eff*_ allowed by the larger number of conformations on which averaging is performed. Overall, this implies smaller model errors for simulations with higher number of effective replicas, where the total model error is computed as $\sigma_{r,i}^{2}=\;\sigma_{i}^{SEM2}+\;\sigma_{r,i}^{B2}$. $\sigma_{i}^{SEM}$ sets the lower limit for the model error and measures the impact of conformational averaging for the *i*th data point. We suggest that the relative error $\sigma_{i}^{SEM}/d_{i}$, where *d*_*i*_ is the *i*th experimental data, can indicate whether the number of effective replicas (and consequently the number of simulated replicas) is sufficient to capture the conformational variability needed to correctly interpret the corresponding data (Supplementary Figure S11). Our results suggest that a relative error lower than 5–10% could be sufficient to achieve a reasonable agreement with the target ensemble. We also note that the relative errors provide information about the sensitivity of different data points to conformational averaging.

These results underlie the importance of using a sufficient large number of replicas in M&M simulations, taking particular care of the number of effective frames in time, which depends on the enhanced sampling technique used, including the employed CVs, the investigated system as well as the specific experimental observable.

## Conclusion

Reliability of MD simulations depends on their statistical precision and experimental accuracy. M&M aims to achieve both by coupling enhanced sampling and Bayesian Inference. Here, we assessed the performance of different MetaD setups, optionally coupled with Metainference, using as test system chignolin. Chignolin is a 10 residues peptide that is able to populate three different conformational states with diverse degrees of compactness and folding thus representing a simple but realistic test case.

In order to assess the statistical precision achievable by diverse enhanced sampling simulations we run three independent replicates for three different Metadynamics setups, either employing PBMetaD or traditional MetaD coupled with multiple walkers and using different combinations of CVs. We showed that block averaging is a robust technique to estimate statistical error, being always a slight overestimation of the standard error computed from the comparison of the triplicates. Still, we observed quite strong deviations in the population values when compared among replicates, suggesting that quantitative conclusions should be drawn with care from a single simulation. Importantly, when using averages calculated over the triplicates, we found an optimal agreement among the different setups, both concerning the free-energies and the population estimates. This quantitative agreement is maintained also with an independent reference simulation (Piana et al., 2020), performed with simulated annealing. Thus, as long as the simulations are well converged and possibly properties are evaluated as averages over independent copies of the simulations, the choice of the enhanced sampling technique does not influence the overall results. These observations support the idea that performing replicates, even if expensive, should become a more common practice, in particular when statistical precision is a core message.

Experimental accuracy can be obtained by Metainference *via* the introduction of restraints toward a set of experimental data. Different issues could affect the success of Metainference simulations, including the quality and quantity of experimental data (Löhr et al., 2017) and the quality of the forward model, as also discussed in this special issue (Ahmed et al., 2021). Here, we highlighted how the combination of MetaD and Metainference (M&M) could create an additional issue related to the number of effective replicas. In Metainference, to restrain the simulation, the experimental data are compared with the same back-calculated observables, averaged over the replicas: this is done to account for the conformational heterogeneity of the system. Nevertheless, the coupling with MetaD, while helping in accelerating the sampling and achieving better statistical precision, could reduce the number of effective replicas (*NR*_*eff*_) on which this averaging is performed. Indeed, MetaD modulates the relative weights of the replicas, where some of them are found in low-energy areas (high probability) and other are in high energy regions (low relative weight). In this work, by performing M&M simulations with either PBMetaD or traditional MetaD setup and using 10 or 100 replicas, we showed how the number of effective replicas is extremely relevant for the reconstruction of conformational ensembles. A too small *NR*_*eff*_ leads to distortions of the prior ensemble that are very far from the desired target. To keep this effect under control we suggest monitoring the relative error caused by $\sigma_{i}^{SEM}$. The latter represents the statistical error we introduce when trying to capture the conformational heterogeneity underlying an experimental observable with a finite number of replicas. Also, we showed that in the context of M&M, PBMetaD could be preferred to traditional MetaD, as it results in a milder reduction in the number of actual replicas. Indeed, the number of replicas should be high enough to capture the conformational heterogeneity of the system as detected by an experimental observable while also compensating to the loss of effective frames resulting from the combination of Metainference with Metadynamics.

Concluding, enhanced sampling techniques and integrative techniques can generate precise and accurate conformational ensembles. Here we showed that well established enhanced sampling techniques provide robust results in particular when performing multiple independent simulations. Moreover, we improve our understanding of Metainference by suggesting how to optimally chose the number of simulated replicas needed to describe correctly the conformational heterogeneity of an ensemble.

## Data Availability

The raw data supporting the conclusion of this article will be made available by the authors, without undue reservation.
