# Supplementary material for: How to Determine Accurate Conformational Ensembles by Metadynamics Metainference: A Chignolin Study Case
Source: Front Mol Biosci. 2021 May 26;8:694130. doi: 10.3389/fmolb.2021.694130 (PMC8187852; doi:10.3389/fmolb.2021.694130)
Supplement: Supplementary file 1 [file DataSheet1.pdf]

Supplementary Material

1 Supplementary Figures

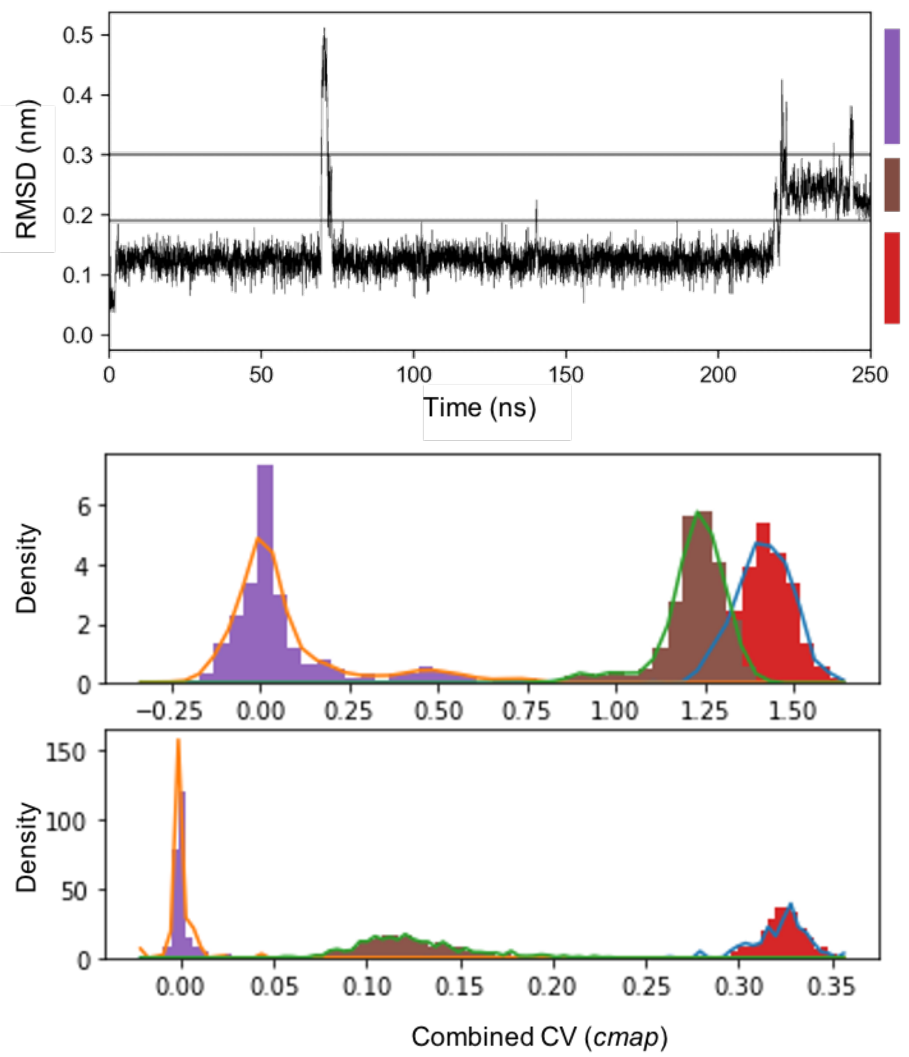

Supplementary Figure 1. *Top panel*: RMSD as a function of the simulation time for a preliminary 250 ns long simulation of chignolin. Three main conformational states are identified: folded (red, RMSD ≤ 1.9 Å), misfolded (brown, 1.9 Å ≤ RMSD ≤ 3.0), unfolded (violet, RMSD > 3.0 Å). The RMSD was computed over the main chain plus the Cβ atoms with respect to a reference folded state of chignolin. *Bottom panel*: distribution of the values for the CV *cmap*, for each of the three identified states, before and after the optimization procedure described in the main text (section 2.2). The *cmap* CV is a combination of contacts among the center of the backbone of i-i+3 amino acids.

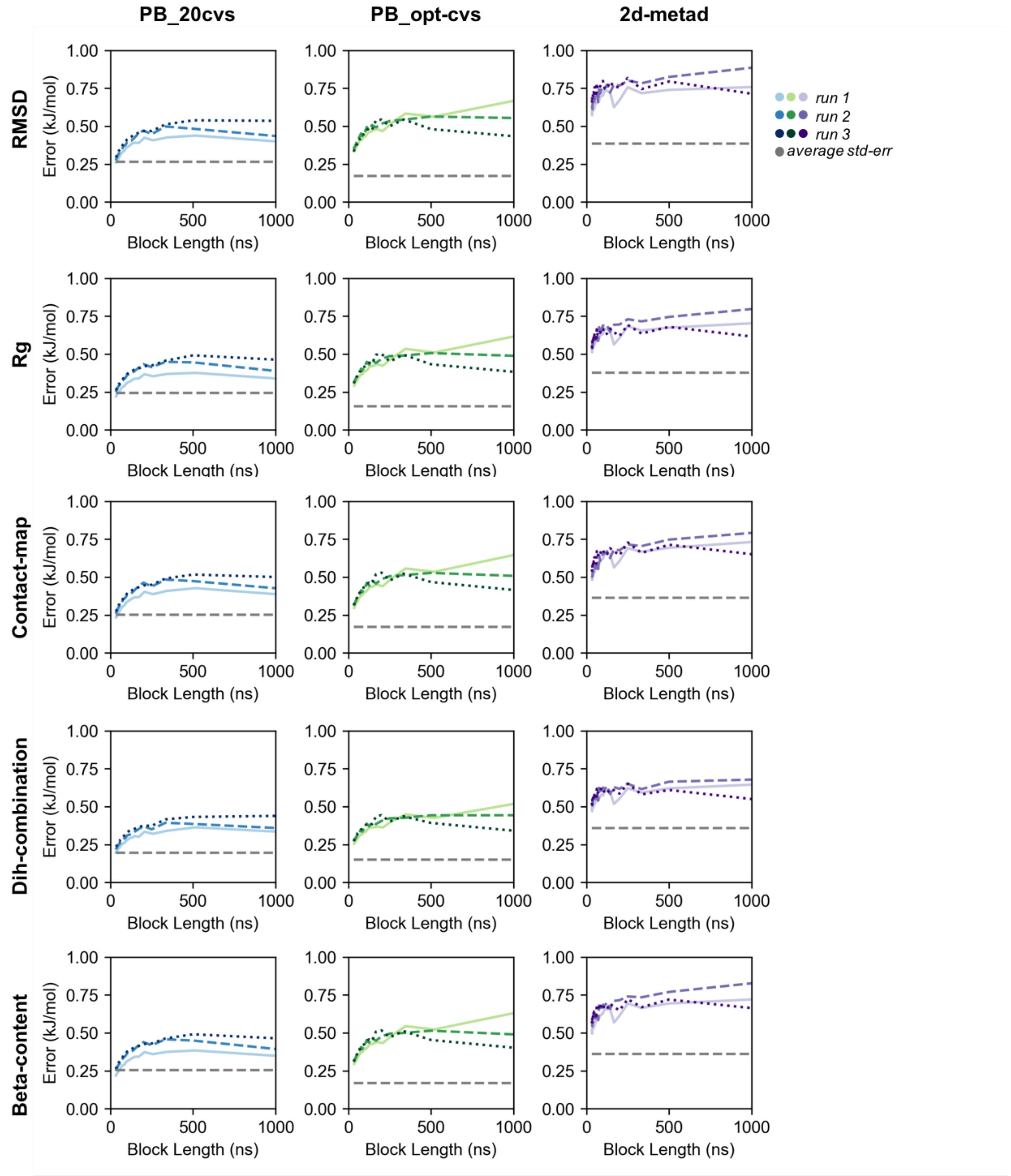

Supplementary Figure 2. Block average analysis performed on 5 different CVs, including RMSD, gyration radius (Rg), contact-map (cmap), dihedral combination (back) and antiparallel beta sheet-content. For each simulation (PB20, PB4 and ME2) are reported the block analysis for each replicate (colored lines) and the average standard-error deriving from the comparison of the triplicates (grey line). Block lengths were chosen to be a fraction (1/30 to 1) of the length of the replica

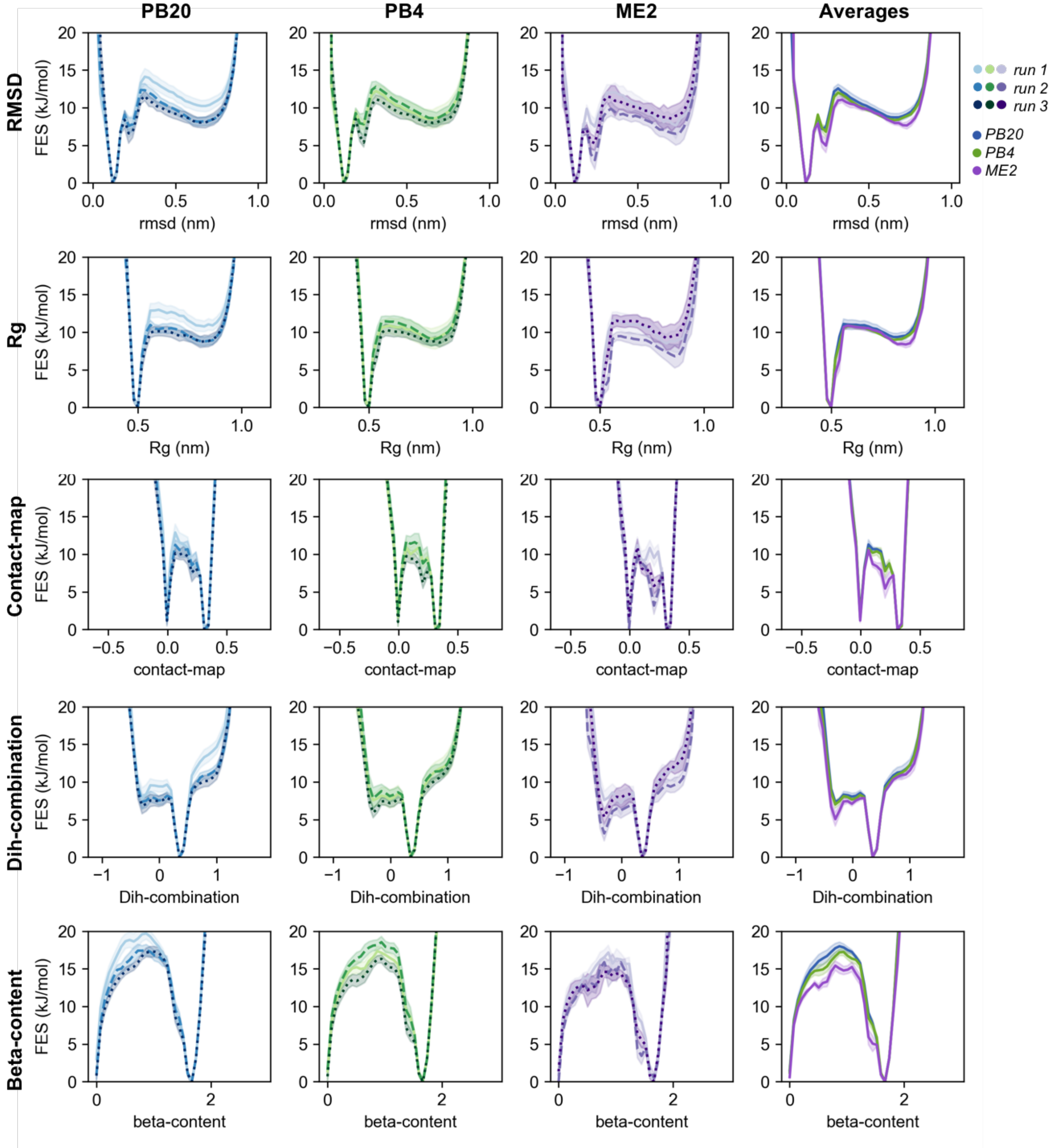

Supplementary Figure 3. Free energies on 5 different CVs, including RMSD, gyration radius (Rg), contact-map (cmap), dihedral combination (back) and antiparallel beta sheet-content. For each simulation are reported the free energies of each replicate (PB20 in blues, PB4 in greens, ME2 in violets shades). The errors are estimated via block-average analysis. In the rightest panels are reported the averages computed over the triplicates for each set of simulations; here the errors are determined as standard errors over the triplicates as described in the main text. In all the pictures the free energies are shifted to set their minimum to 0.

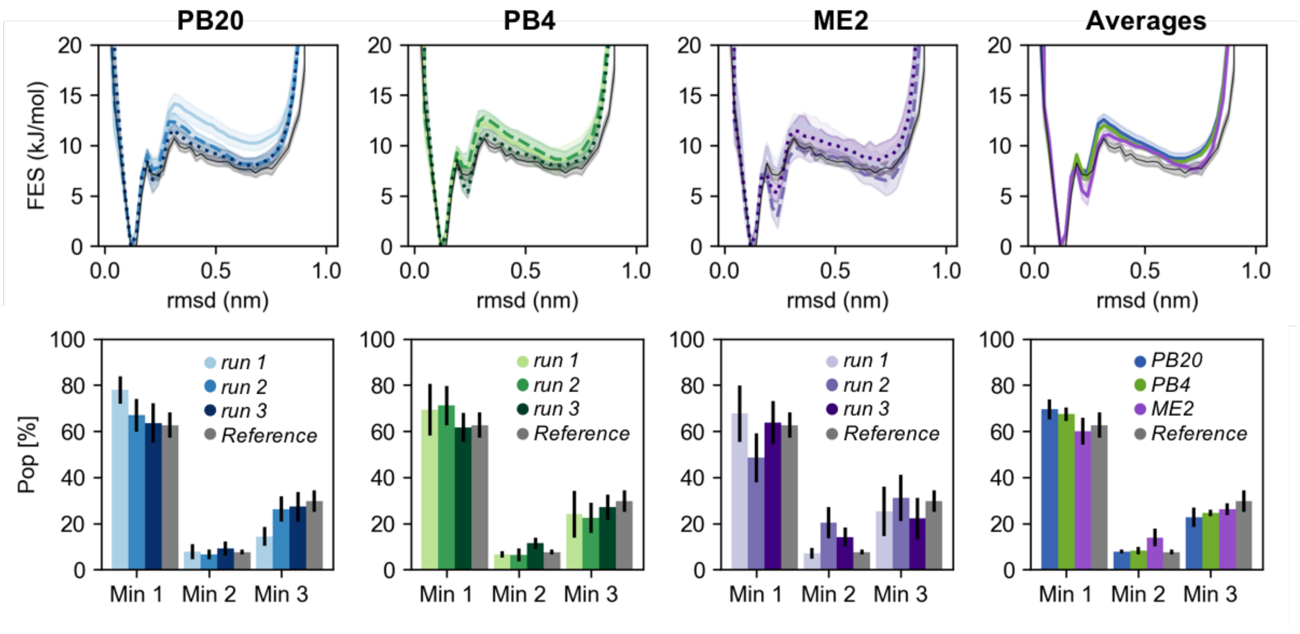

Supplementary Figure 4. The RMSD free energies (top panels) and the population of the three main chignolin minima (bottom panels) are represented for different sets of simulations (PB20 in blues, PB4 in greens, ME2 in violets shades). The different shades indicate each of the runs of the triplicates. The grey lines/bars represent the results from the reference DES-amber 40 μs long simulated tempering simulation of chignolin by Piana et al. (Piana et al. 2020). The errors are estimated via block-average analysis. In the rightest panels are reported the averages computed over the triplicates for each set of simulations; here the errors are determined as standard errors over the triplicates as described in the main text. In all the pictures the free energies are shifted to set their minimum to 0.

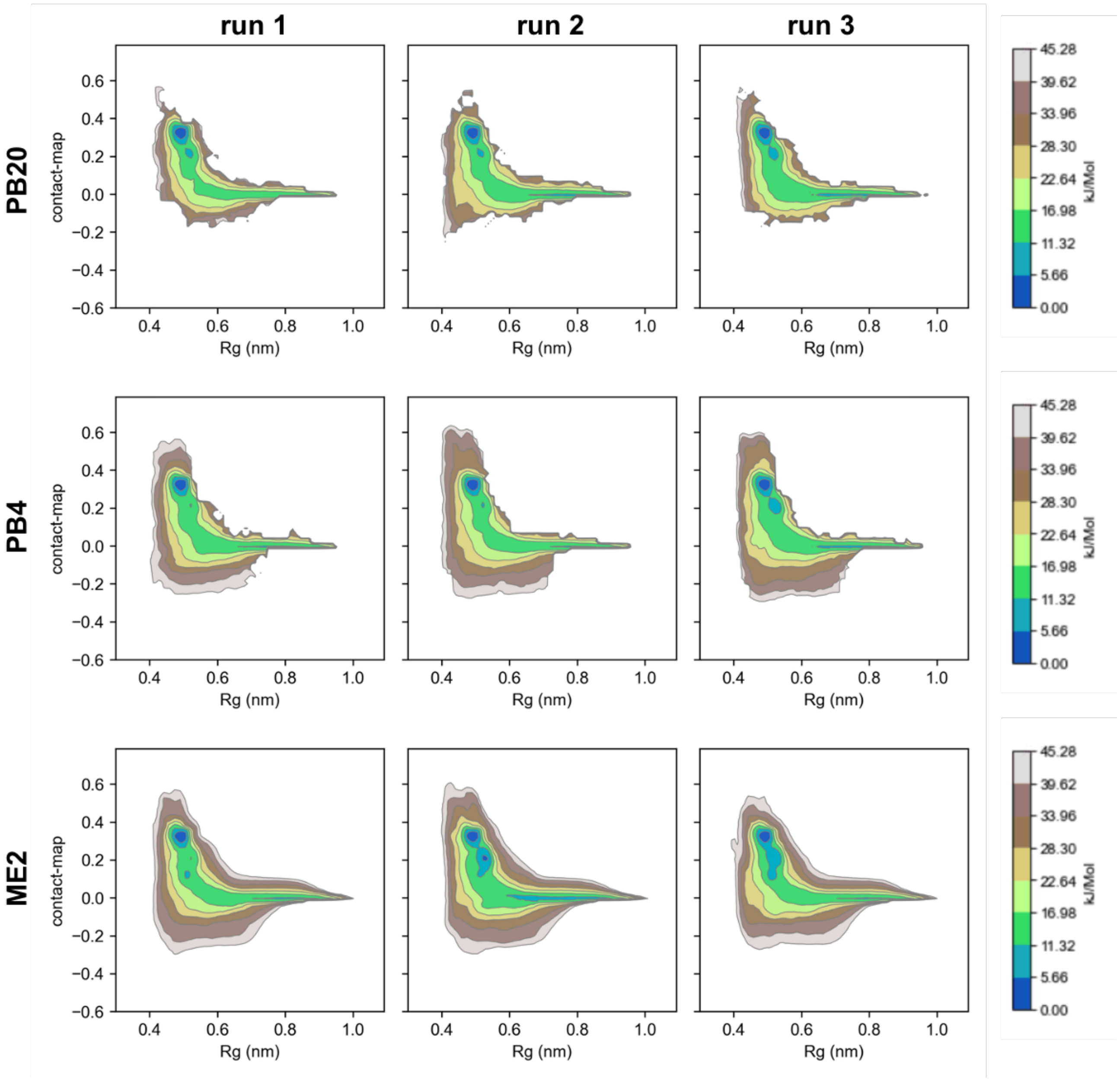

Supplementary Figure 5. 2D-free energy surface of chignolin as a function of the gyration radius (Rg) and the optimized variable cmap. The free energies are reported for each replicate of the PB20, PB4 and ME2 simulations.

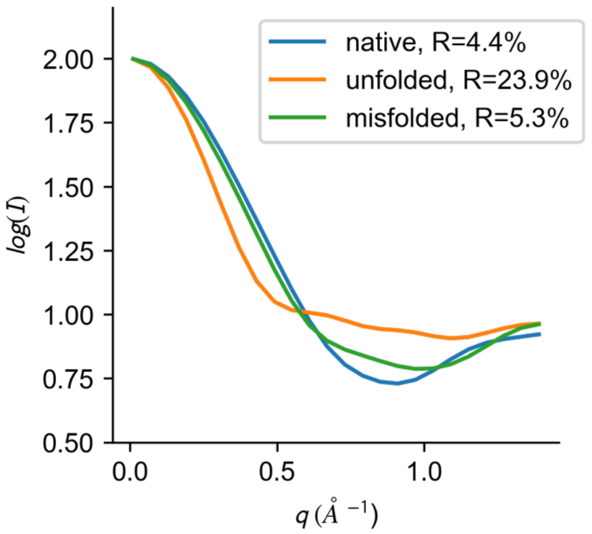

Supplementary Figure 6. Synthetic SAXS profiles for the three conformational states of chignolin represented in Figure 1 (i.e., native, unfolded, and misfolded). For each profile it is reported the R-factor, reporting on their distance from the reference SAXS profile derived from the DES-amber simulation (I_{ref}). The R-factor is computed as: $R_{factor} = \langle \left| \frac{I - I_{ref}}{I_{ref}} \right| \rangle * 100$, where I is the scattering intensities of the analyzed profile and $\langle \ldots \rangle$ is the average performed over the 24 considered equally spaced scattering angles.

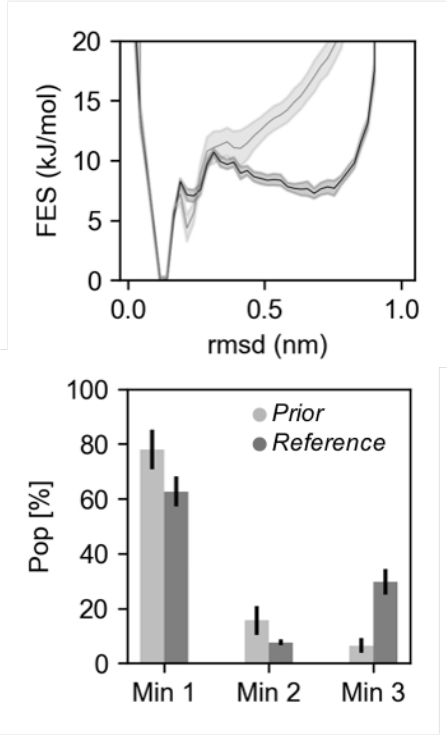

Supplementary Figure 7. RMSD free energies (top panel) and population of the three main chignolin minima (bottom panel) for the prior amber99sb-ildn simulation (light grey) and the reference DES-amber 40 μs long simulated tempering simulation of chignolin (dark grey). The errors are estimated via block-average analysis. The free energies are shifted to set their minimum to 0.

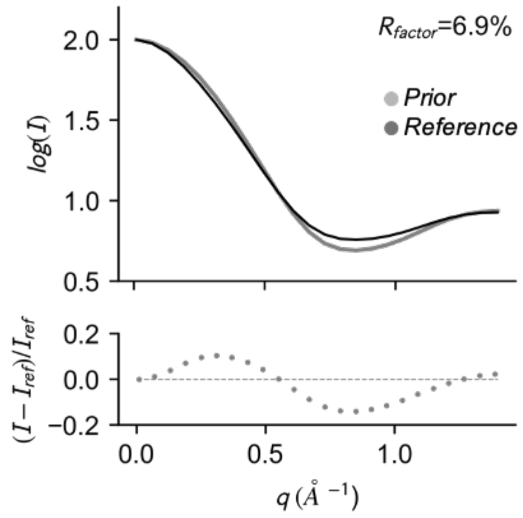

Supplementary Figure 8. Top panel: SAXS profiles back-calculated from the prior amber99sb-ildn simulation (light grey) and the reference DES-amber simulated tempering simulation of chignolin (dark grey). In the bottom panel are reported the deviations $\frac{I-I_{ref}}{I_{ref}}$ for each scattering angle. The computed $R_{factor} = \langle \left| \frac{I-I_{ref}}{I_{ref}} \right| \rangle * 100$, reporting on the difference between the two back-calculated profiles, is 6.9%.

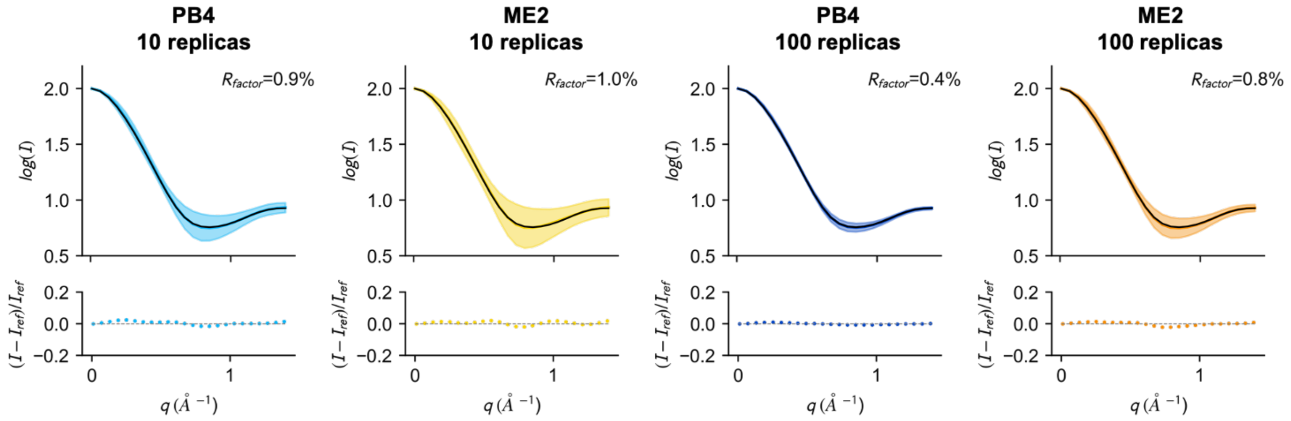

Supplementary Figure 9. For each simulation (PB4 and ME2, with either 10 or 100 replicas), are reported: (top panel) the back-calculated SAXS profile, in which the shade indicates the error estimated via Metainference, compared with the profile caclulated from the reference DES-amber simulation; (bottom panel) the deviations $\frac{I-I_{ref}}{I_{ref}}$ for each scattering angle. Here the ranges of the y-axis are maintained as in Figure S8 to allow comparison with SAXS profiles back-calculated from the prior amber99sb-ildn simulation. The computed $R_{factor} = \langle\left|\frac{I-I_{ref}}{I_{ref}}\right|\rangle * 100$ with respect to the reference DES-amber simulation are reported and range from 0.4% to 1.0%.

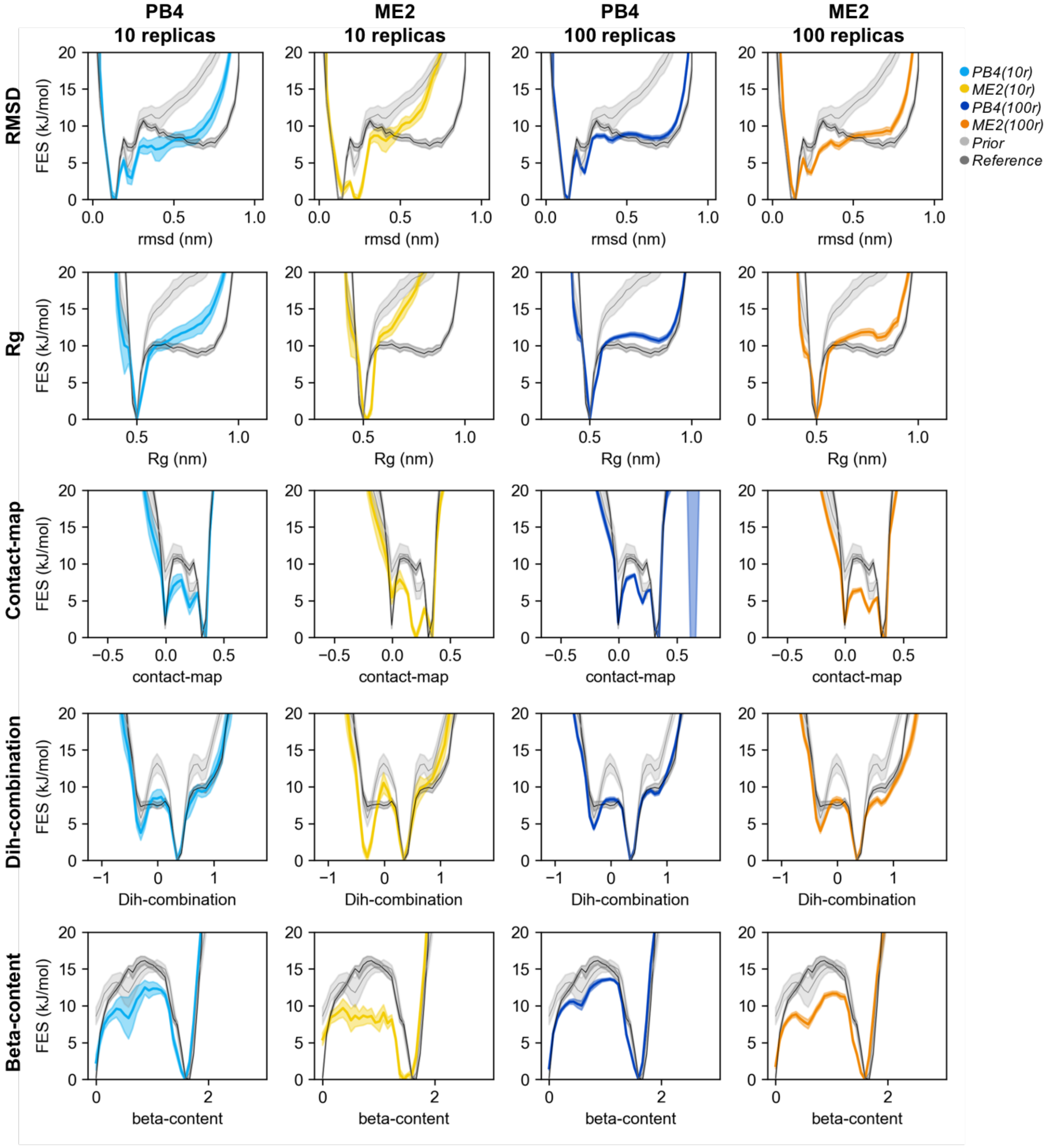

Supplementary Figure 10. Free energies on 5 different CVs, including RMSD, gyration radius (Rg), contact-map (cmap), dihedral combination (back) and antiparallel beta sheet-content. For each simulation (PB4 and ME2, with either 10 or 100 replicas), the free energy of the simulation is compared with the ones of the prior amber99sb-ildn simulation (light grey) and the reference DES-amber 40 μs

long simulated tempering simulation of chignolin (dark grey). The errors are estimated via block-average analysis. In all the pictures the free energies are shifted to set their minimum to 0.

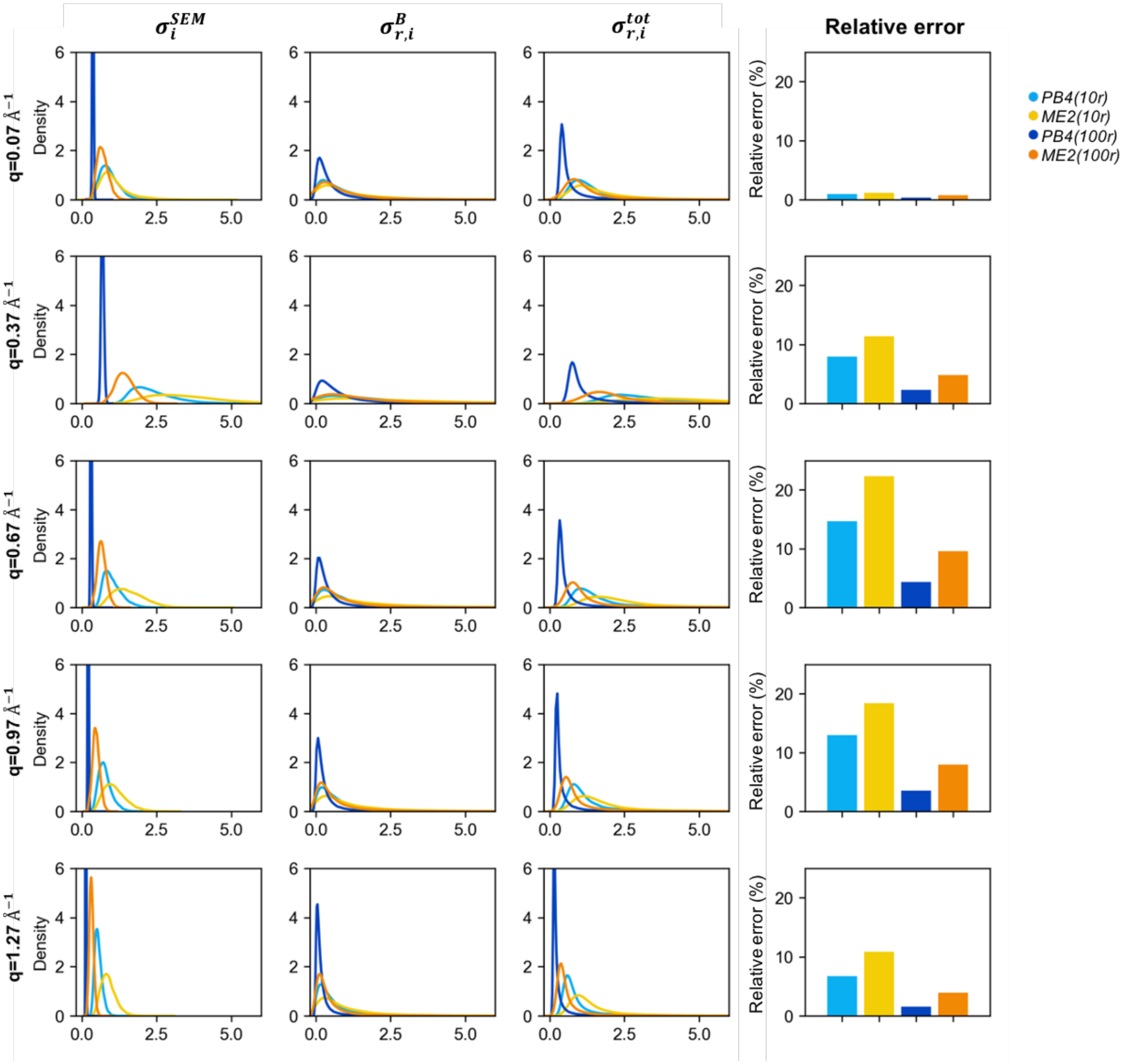

Supplementary Figure 11. Probability density functions of the uncertainty parameters σ_i^{SEM}, $\sigma_{r,i}^{B}$ and $\sigma_{r,i}^{tot}$ (expressed in a.u.) for the 5 representative scattering angles equally spaced in the range 0.01-1.39 Å^{-1}. In the rightest panels are reported the relative errors associated to σ_i^{SEM} (computed as: σ_i^{SEM}/d_i, where d_i is the ith experimental data).
